# Complete genome sequence of *Halomonas litopenaei* YBW-3-4-1, a polyhydroxyalkanoate-producing halophile

**DOI:** 10.1128/mra.01104-25

**Published:** 2026-02-25

**Authors:** Sondor Ganbat, Yujin Son, Dariimaa Ganbat, Seong-Bo Kim, Sung Tae Kim, Mi Hwa Park, Sang-Jae Lee

**Affiliations:** 1 Department of Bioscience and Research Center for Extremophiles and Marine Microbiology, Silla University 65486, Busan, South Korea; 2 Department of Integrative Biotechnology (IBT), Yonsei University 26721, Incheon, South Korea; 3 Bio-Living Engineering Major, Global Leaders College, Yonsei University 26721, Seoul, South Korea; 4 Department of Nanoscience and Engineering, Inje University 26719, Gimhae, South Korea; 5 Department of Pharmaceutical Engineering, Inje University 26719, Gimhae, South Korea; 6 Department of Food and Nutrition, College of Human Ecology, Silla University 65486, Busan, South Korea; Queens College Department of Biology, Queens, New York, USA

**Keywords:** *Halomonas litopenaei*, polyhydroxyalkanoates (PHAs), genome sequencing, halophilic bacteria

## Abstract

We report the complete genome sequence of *Halomonas litopenaei* strain YBW-3-4-1, a polyhydroxyalkanoate (PHA)-producing bacterium isolated from seawater in South Korea. The 4.93 Mb circular chromosome was assembled using PacBio and Illumina sequencing, revealing 4,294 predicted genes, including pathways for PHA biosynthesis and regulation, supporting its potential in sustainable bioplastic production.

## ANNOUNCEMENT

Plastic pollution remains a major challenge to sustainable development (1, 2). Biodegradable polymers such as polyhydroxyalkanoates (PHAs) are promising alternatives to petroleum-based plastics (3–5). PHAs are synthesized by diverse microorganisms as intracellular carbon and energy reserves and are degradable in terrestrial and marine environments (3, 4, 6). The genus *Halomonas* has attracted attention for its robust metabolism, ability to utilize diverse feedstocks, and high salt tolerance, which minimize contamination risk (4, 5, 7). Here, we report the complete genome sequence of *Halomonas litopenaei* strain YBW-3-4-1, a PHA-producing bacterium.

Strain YBW-3-4-1 was isolated from seawater collected near the Yeongi Breakwater, Tongyeong, South Korea (34°52′24.24″ N, 128°28′04.93″ E). The sample was serially diluted in 0.85% saline buffer, spread onto Marine agar 2216 (BD Difco), and incubated aerobically at 37°C. Colonies were purified by repeated streaking, and pure cultures were cryopreserved in 5% DMSO at −80°C. The 16S rRNA gene was amplified with universal primers 27F and 1492R (8), and analyzed by BLAST against the NCBI database, showing 99.93% identity to *Halomonas* sp. strain DN3 (GenBank CP099965.1).

A single colony was cultured overnight in Marine Broth at 37°C. Genomic DNA was extracted using the Wizard Genomic DNA Purification Kit (Promega), sheared with a g-TUBE (Covaris), and purified using AMPure PB magnetic beads (Beckman Coulter). A PacBio SMRTbell library was prepared using the SMRTbell Prep Kit 3.0 (Pacific Biosciences) and sequenced on the PacBio Sequel II platform. PacBio HiFi reads were generated using SMRT Link v13.0.0.207600 with default parameters, performing read quality control, error correction, and adapter trimming. Sequencing yielded 47,066 reads totaling 383,856,161 bp, with a mean read length of 8,155 bp and an *N*_50_ of 9,278 bp (77× coverage). To improve assembly accuracy, an Illumina TruSeq Nano paired-end library (2 × 150 bp) was sequenced on the NovaSeq 6000 platform, generating 17,219,404 reads comprising 2,600,130,004 bp (373× coverage). Illumina reads were adapter-trimmed and quality-filtered with Trimmomatic v0.38 (9).

PacBio reads were assembled *de novo* using the Microbial Assembly application in SMRT Link v13.0.0.207600 (HGAP v4) (10), which identified and trimmed overlapping contig termini and rotated the circular contig to a predicted origin of replication. The assembly was polished with Illumina reads using Pilon v1.21 (11), resulting in a single circular chromosome of 4,934,047 bp with a GC content of 68%. Genome completeness was assessed using BUSCO v5.1.3 (12) with 100.0% complete single-copy BUSCOs. Default parameters were used for all software unless otherwise specified.

Annotation with the NCBI Prokaryotic Genome Annotation Pipeline v6.10 predicted 4,294 genes, including 4,211 coding sequences, 64 tRNA genes, and 15 rRNA genes (5S, 16S, and 23S). Genes associated with PHA biosynthesis (*phaC*, *phaA*, *phaB, phaP*, *phaR,* and *phaZ*) were identified (13), supporting the strain’s potential for PHA production.

## Data Availability

The whole genome has been deposited in GenBank under accession number CP197403. The genome sequence is associated with BioProject number PRJNA1299619 and BioSample number SAMN50309239. Raw sequencing data are available in the Sequence Read Archive (SRA) under accession numbers SRX29957756 and SRX29976897. The 16S rRNA gene sequence has been deposited under accession number PX204999.
